# Cancer incidence in a cohort of Swedish merchant seafarers between 1985 and 2011

**DOI:** 10.1007/s00420-021-01828-2

**Published:** 2022-01-08

**Authors:** Karl Forsell, Ove Björ, Helena Eriksson, Bengt Järvholm, Ralph Nilsson, Eva Andersson

**Affiliations:** 1 Occupational and Environmental Medicine, Sahlgrenska Academy, Gothenburg University, Gothenburg, Sweden; 2 Department of Radiation Science, Oncology, Umeå University, Umeå, Sweden; 3 Department of Public Health and Clinical Medicine, Section of Sustainable Health, Umea University, Umea, Sweden

**Keywords:** Cohort, Follow-up, Neoplasm (epidemiology), Occupational disease (epidemiology), Risk factor, Cancer incidence, Seafarer, Sweden, Occupational exposure, Lifestyle, Record linkage

## Abstract

**Purpose:**

Lung cancer, mesothelioma and several lifestyle-associated cancer forms have been reported more common in merchant seafarers. However, few studies reflect recent occupational settings and women seafarers are usually too scarce for meaningful analyses. We conducted a study on cancer incidence between 1985 and 2011 in a Swedish cohort consisting of male and female seafarers.

**Methods:**

All seafarers in the Swedish Seafarers’ Register with at least one sea service between 1985 and 2011 and a cumulated sea service time of ≥ 30 days (*N* = 75,745; 64% men, 36% women; 1,245,691 person-years) were linked to the Swedish Cancer Register and followed-up until 31 December 2011. Standardized incidence ratios (SIR) were calculated with the general population as reference.

**Results:**

There were 4159 cancer cases in total, with 3221 among men and 938 among women. Male seafarers had an increased risk of total cancer (SIR 1.05; 95% CI 1.01–1.09), lung cancer (SIR 1.51; 95% CI 1.35–1.67) and urinary bladder cancer (SIR 1.17; 95% CI 1.02–1.33). Several lifestyle-associated cancer forms were more common in men. Previous work on tankers was associated with leukaemia (SIR 1.41; 95% CI 1.00–1.86). The risk of cancer decreased with a start as a male seafarer after 1985, with a significant trend for total cancer (*P* < 0.001), lung cancer (*P* = 0.001) and, for tanker seafarers, leukaemia (*P* = 0.045). Women seafarers had an increased risk of lung cancer (SIR 1.54; 95% CI 1.23–1.87) but the risk of total cancer was not increased (SIR 0.83; 95% CI 0.78–0.89).

**Conclusions:**

In this cohort of merchant Swedish seafarers 1985–2011, the risk of total cancer was increased in men but not in women compared to the general population. Lung cancer was increased in both genders. The risk of cancer seems to decrease over the last decades, but better exposure assessments to occupational carcinogens and longer observation times are needed.

## Background

In 1998, a Swedish epidemiological cancer study on Swedish male merchant seafarers from national census, found an increased risk of total cancer by 29% (Standardized Incidence Ratio, SIR, 1.29; 95% confidence interval, CI 1.22–1.39) (Nilsson 1998). The reference group consisted of all economically active men in Sweden. Lung cancer and mesothelioma were more common, especially among the engine room crewmembers. Other epidemiological studies, most performed on Nordic seafarers, have shown similar results together with increased risks of cancer of the urinary bladder, stomach, lip, skin, pancreas and the prostate (Greenberg 1991; Pukkala and Saarni 1996; Saarni et al. 2002; Rafnsson and Sulem 2003; Pukkala et al. 2009). There are several occupational factors present in seafaring that may explain an increased risk of cancer. Asbestos, from the ships interior or cargo, may cause pleural mesothelioma and lung cancer in seafarers (IARC 1987, 2012; Saarni et al. 2002; Forsell et al. 2007, 2017; Ugelvig Petersen et al. 2018). Exposures to polyaromatic hydrocarbons (PAHs) present in soot, exhausts and in contaminated oils may be involved in the aetiology of bladder cancer, lung cancer and skin cancer (IARC 1984a, 1984b, 2006, 2013; Moen et al. 1996; Boffetta et al. 1997, 2001; Nilsson et al. 2004; Kaerlev et al. 2005; Forsell et al. 2007; Olsson et al. 2010). The sun’s ultraviolet radiation (UV radiation) is a carcinogenic exposure when working outside on a ship, with increased risks of skin and lip cancer (Pukkala et al. 2009; Oldenburg et al. 2015; IARC 2018; Ugelvig Petersen et al. 2018). Certain lifestyle-associated factors of cancer may be more prominent among seafarers, such as tobacco smoking, alcohol use and infections (Greenberg 1991; Pukkala and Saarni 1996; Kaerlev et al. 2005).

A special group of interest has been seafarers working on tankers, where important exposures to the carcinogen benzene have been demonstrated (Moen et al. 1993, 1995; Nilsson et al. 1996; Forsell et al. 2019). Furthermore, tanker crewmembers have shown increased risks of leukaemia and other blood malignancies (Nilsson et al. 1998; Saarni et al. 2002; Forsell et al. 2020), indicating a carcinogen effect from such occupational benzene exposure.

An eventual effect on cancer incidence from ship modernization and an improved safety climate, especially for Nordic seafarers, is not known. Examples of such are the step-wise reduction of threshold limit values for several carcinogenic chemicals (IMO 1974, 2000, 2003, 2011) and the introduction of closed handling of chemical cargoes (Mowe et al. 1977; Moen et al. 1988; Williams et al. 2005; Kirkeleit et al. 2006; Forsell et al. 2019).

Cancer among women seafarers is seldom reported since the population has been limited in numbers, although their number has increased in Nordic countries to actually represent around 20–30% of the work force (Kaerlev et al. 2005; Forsell et al. 2017). A Danish study with a follow-up of 1986–1999 reported increased incidences compared to the general female population for total cancer and for the following sites: pharynx, oesophagus, rectum, liver, gallbladder and bile ducts, lung (including bronchus and trachea), cervix uteri and ovaries (Kaerlev et al. 2005). An older Finnish study also found higher cancer incidence among women seafarers from all sites, mouth and pharynx, larynx, lung and bronchus and cervix uteri and breast cancer (Pukkala and Saarni 1996).

The aims of the present study were to investigate any change in cancer incidence among Swedish merchant seafarers between 1985 and 2011, and to identify any groups of seafarers by gender or work category at special risk of cancer.

## Materials and methods

This longitudinal cohort study on Swedish seafarers was enabled through matching the Swedish Seafarers Register (SR) with the Swedish Cancer Register (CR). All Swedish seafarers have to be registered in the SR. The SR is administered by the Swedish Transport Agency, and it holds information on seafarer’s time and length of sea services, type of ship the service took place on and the seafarer’s position on board. Our initial cohort consisted of all seafarers with any registered sea service from 1985, corresponding to the start of the digitization of the SR, up to 2013 (*n* = 105,098). However, since information on any sea service before 1985 had during the digitization been manually entered by the Authority (only for seafarers in service at that time), we could consider even this data when assessing the cumulative sea service time per seafarer. We excluded non-Swedish citizens (*n* = 9536), seafarers deceased before 1985 (*n* = 101), with re-used PINs (*n* = 50) or born before 1920 (*n* = 198). The last group was excluded since they would have been > 65 years, the usual retirement age, when the observation period started. Seafarers with a cumulative service time of < 30 days were also excluded (*n* = 7223) to avoid short-term workers at sea. Finally, since we only got data on cancer up to 2011 from the CR at the start of the study, seafarers with sea services only between 2011 and 2013 were excluded (*n* = 12,245).

Among those 75,745 included in the cohort, 21 582 had their first sign on before 1985. There were about 20,000 cohort members in the register each year, with a maximum of 25,693 registered in 1992, then slowly decreasing. We used the definition of a merchant ship as any vessel that transports cargo or carries passengers. Seafarers could have worked on ships of any flag. The number of ships sailing the Swedish flag has diminished a lot from the 70’ies until now. However, there has been a relatively stable numbers of registered seafarers during 1985 to 2011.

The CR is hold by the National Board of Health and Welfare and started in 1958. Physicians are obliged to report any new malignancy to the register. Tumours from CR were classified according to the International Classification of Diseases (ICD-7). The Swedish Transport Agency sent the SR data to Statistics Sweden for data on migration or other vital events, and thereafter to the National Board of Health and Welfare for linkage with the CR. Record linkage was done by the subjects’ personal identity number (PIN). All data were anonymised by the Board before transferral to us.

### Statistical analysis

The seafarers were classified by sea service position as ‘ever deck rating’, ‘ever deck officer’, ‘ever engine rating’, ‘ever engine officer’ and ‘ever catering/service’. We also analysed cancer and ‘ever work on tankers’. In the analyses, we used a minimum of 30 days of cumulated service time per such work category.

Person-years at risk were calculated starting from 1 January 1985, age of 20 years or first time of sea service in SR if later and until first emigration, time of death, age of 85 years or end of follow-up 31 December 2011. The person-years were stratified by gender, 5-year age groups and 1-year calendar periods. The expected number of cancer cases for these strata was calculated using the general Swedish population as reference. SIR was then achieved by dividing the observed by the expected number of cancer cases with a 95% CI. For the CI, we assumed a Poisson distribution for the number of observed cancer cases. Data on cancers were analysed when the number of observed cases was > 3. Age and cumulated time of sea services are given with the median with its first and third quartiles (Q1 and Q3, respectively).

SIRs were calculated for the whole follow-up and for three different calendar periods, each defined by the first registered sea service of each seafarer (< 1985; 1985–1991; ≥ 1992). We tested for trend between different SIRs of the three calendar periods using Chi^2^ test of trend as described by Breslow and Day (Breslow and Day 1987). Further analyses included a 10 years’ latency, a cumulative sea service time of 5 years or more and haematologic malignancy among seafarers who had worked on tankers. The last analysis was restricted to men since few women had worked on tankers. All analyses were made using R 3.2.2 and SAS 9.4.

The study was approved by the Regional Ethical Committee of Gothenburg (193–13).

## Results

The final cohort consisted of 75,745 seafarers, 36% women, 64% men, representing in total 1,245,691 person-years at risk (Table 1). Age at start of follow-up in three age spans are given in Table 1. The median age at the end of the observation period was 45 years (Q1 35 years; Q3 56 years), which was similar for both sexes (data not shown). The median employment time of a seafarer was 4 years (Q1 1 year; Q3 15 years), with a median of 7 years for men and 2 years for women (further information in Table 1). During the observation period, 6835 (9%) emigrated and 4876 (6%) died.

**Table 1:** Characteristics (selected) of the cohort of men and women Swedish seafarers between 1985 and 2011

	Total Person-years		Men	Women
			Person-years	Person-years
Person-years for all seafarers	1,245,691		823,523	422,168
Start < 1985	510,228		409,402	100,827
Start 1985–1991	414,350		214,096	200,254
Start > 1992	321,113		200,025	121,087
	*n*		*n*	*n*
Total numbers of seafarers	75,745		48,787	26,958
Position^a^				
Deck				
Total	28,211		24,915	3296
Officers	9261		8858	403
Ratings	26,200		23,079	3121
Engine				
Total	12,202		11,924	278
Officers	5940		5861	79
Ratings	10,761		10,502	259
Catering/service	42,300		17,949	24,351
Tankers^a^				
Total	14,596		13,197	1399
Deck	8475		8061	414
Vital status 31st Dec 2011				
Alive	64,034		40,473	23,561
Dead	4876		4167	709
Emigrated	6835		4147	2688
Age at start of follow-up				
< 30 years	48,404		28,146	20,258
30–59 years	26,240		19,748	6492
≥ 60 years	1,101		893	208
Time first to last sea service until 2011				
< 1 year	17,419		9518	7901
1–5 year	23,075		12,456	10,619
5–10 years	10,173		6176	3997
≥ 10 years	25,078		20,637	4441

^a^Analysis based on “ever” having held such a position (overlap possible)

Fifty-one percent of male seafarers had ever worked in the deck department, 24% in the engine department and 37% in the catering/service department (Table 1). Of women, 90% had ever worked in the catering or service department. Apart from catering many of the seafarers had worked at different positions and ships. Twenty-seven percent of men, but only 5% of women, had ever worked on tankers. Eighty percent of women and 40% of men had only worked on passenger ships. Besides passenger ships, the most common ships were cargo ships. Twenty-eight percent of all seafarers had had their first registered sea service before 1985, 26% between 1985 and 1991 and 46% in 1992 or later (data not shown).

There were 4,159 cases of cancer in the cohort. The majority of cancer cases, 71%, had occurred among seafarers with their first registered sea service before 1985, 18% with first sea service 1985–1991 and 11% in 1992 or later (data not shown).

### Cancer in male seafarers

The incidence of total cancer among male seafarers was increased (Table 2). A SIR of 1.15 was noted for deck officers. Cancers of the lung, the urinary bladder, mouth/pharynx, oesophagus and the larynx were all more common than in the general population (Table 3). Lung cancer was more common in all departments or rank, with the most elevated SIR for engine ratings (Table 2). Malignant melanoma and other skin cancer were not significantly increased. Neither was the incidence of cancer of the tongue, stomach, liver, colon/rectum nor pancreas (Table 3).

**Table 2:** Standardized Incidence Ratio (SIR) for total cancer and lung cancer in Swedish seafarers 1985–2011 per position (”ever”) and gender

	Men			Women		
	Obs	SIR (95% CI)		Obs		SIR (95% CI)
Total cancer						
All positions	3 221	1.05 (1.01–1.09)		938		0.83 (0.78–0.89)
Deck*						
Officers	1 137	1.15 (1.08–1.21)		27		1.15 (0.73–1.62)
Ratings	1 717	1.08 (1.03–1.13)		51		0.73 (0.54–0.93)
Engine^a^						
Total	1 066	1.07 (1.00–1.13)		4		0.67 (0.17–1.33)
Officers	691	1.06 (0.98–1.14)		≤ 3		(expected 2)
Ratings	903	1.12 (1.05–1.19)		4		0.72 (0.18–1.45)
Catering/service^a^	727	1.04 (0.96–1.11)		880		0.83 (0.77–0.88)
Tankers, all positions	941	1.08 (1.01–1.15)		65		0.94 (0.72–1.17)
Lung cancer						
All positions	341	1.51 (1.35–1.67)		84		1.54 (1.23–1.87)
Deck^a^						
Officers	108	1.38 (1.12–1.65)		4		3.07 (0.77–6.14)
Ratings	186	1.57 (1.35–1.79)		≤ 3		expected 3
Engine^a^						
Total	125	1.64 (1.36–1.93)		0		expected 0
Officers	76	1.49 (1.15–1.84)		0		expected 0
Ratings	111	1.82 (1.49–2.16)		0		expected 0
Catering/service^a^	74	1.58 (1.24–1.95)		81		1.57 (1.24–1.92)
Tankers, all positions	121	1.87 (1.54–2.21)		6		1.68 (0.56–3.08)

*Obs* observed cases; *CI* confidence interval

^a^Analysis based on “ever” having held such a position (overlap possible)

**Table 3:** Standardized Incidence Ratio (SIR) by specific site for cancer 1985–2011 among Swedish seafarers, men and women

ICD-7	Cancer	Men		Women	
		Obs	SIR (95% CI)	Obs	SIR (95% CI)
140	Lip	17	1.63 (0.86–2.50)	≤ 3	
	≥ 5 years cumulative sea service	16	1.96 (1.10–2.94)		
141	Tongue	20	1.25 (0.75–1.81)	≤ 3	
143–148	Mouth, pharynx	76	1.66 (1.29–2.05)	4	0.56 (0.14–1.12)
	≥ 5 years cumulative sea service	66	2.02 (1.56–2.51)		
150	Oesophagus	54	1.52 (1.13–1.94)	< 3	
	≥ 5 years cumulative sea service	45	1.64 (1.17–2.15)		
151	Stomach	78	1.14 (0.89–1.40)	12	0.95 (0.48–1.51)
153	Colon	193	1.01 (0.87–1.15)	50	0.91 (0.68–1.17)
154	Rectum	141	1.06 (0.89–1.25)	26	0.87 (0.57–1.20)
155.0	Liver	34	1.04 (0.70–1.41)	≤ 3	
155.1–9	Gallbladder	14	0.80 (0.40–1.25)	≤ 3	
157	Pancreas	52	0.87 (0.64–1.12)	15	1.00 (0.53–1.53)
160	Nasal cavity	6	1.07 (0.36–1.95)	≤ 3	
161	Larynx	44	1.98 (1.40–2.57)	4	3.10 (0.78–6.20)
	≥ 5 years cumulative sea service	38	2.21 (1.51–2.97)		
162.1	Lung	341	1.51 (1.35–1.67)	84	1.54 (1.23–1.87)
	≥ 5 years cumulative sea service	183	1.61 (1.43–1.80)	43	1.61 (1.16–2.10)
162.2	Pleura (mesothelioma)	16	1.29 (0.73–1.93)		
170	Breast			291	0.74 (0.65–0.82)
171	Cervix uteri			49	0.96 (0.71–1.24)
172	Uterus			27	0.58 (0.36–0.81)
175	Ovaries			46	1.02 (0.73–1.32)
177	Prostate	812	0.92 (0.86–0.98)		
178	Testis	77	1.02 (0.79–1.26)		
179	Male genitals	10	0.90 (0.36–1.54)		
180	Kidney	79	0.86 (0.68–1.06)	18	1.10 (0.61–1.65)
181	Urinary bladder	218	1.17 (1.02–1.33)	12	0.70 (0.35–1.11)
	≥ 5 years cumulative sea service	183	1.26 (1.08–1.44)	8	0.91 (0.34–1.58)
190	Malignant melanoma	176	1.04 (0.88–1.19)	75	0.90 (0.71–1.10)
191	Other skin	177	1.12 (0.95–1.29)	22	0.71 (0.42–1.04)
193	Brain	107	0.95 (0.77–1.13)	48	0.93 (0.68–1.20)
194–195	Thyroid	48	0.76 (0.55–0.98)	35	0.62 (0.43–0.83)
196	Bone	7	1.09 (0.31–2.03)	≤ 3	
197	Connective tissue	24	1.00 (0.63–1.42)	4	0.52 (0.13–1.04)
199	Other	91	1.18 (0.95–1.43)	20	0.78 (0.47–1.13)
200, 202	Non-Hodgkin’s lymphoma	108	0.93 (0.75–1.11)	25	0.91 (0.58–1.27)
201	Hodgkin’s disease	16	0.78 (0.44–1.17)	8	0.96 (0.36–1.68)
203	Multiple myeloma	32	0.82 (0.54–1.13)	8	0.98 (0.37–1.72)
204–209	Leukaemia	105	1.01 (0.82–1.22)	18	0.65 (0.36–0.98)
140–209	All sites	3221	1.05 (1.01–1.09)	938	0.83 (0.78–0.89)
	≥ 5 years cumulative sea service	2491	1.10 (1.06–1.15)	357	0.82 (0.73–0.90)

*Obs* observed cases; *CI* confidence interval

Applying a minimum of 5 years of cumulated sea service to the analyses increased the already elevated SIRs (Table 3). The risk of lip cancer then was significantly increased. Applying a 10 years lag between first sea service and time of diagnosis had no apparent effect on the SIRs other than a small increase in general (Table 4). Comparing incidence of cancer in seafarers with a first sea service before 1985 with seafarers with a later first employment, we found a significant trend of a decreased risk of cancer. This trend was supposedly an effect of a sharp decline in the lung cancer risk (Table 5).

**Table 4:** Standardized Incidence Ratio (SIR) for all and selected cancers among Swedish seafarers, men and women, applying a 10 years’ latency from start of exposure (first sea service) (*n* = 54 926); 1985–2011

ICD-7		Men		Women	
		Obs	SIR (95% CI)	Obs	SIR (95% CI)
	All	2 912	1.06 (1.03–1.10)	741	0.84 (0.78–0.90)
140	Lip	17	1.79 (0.95–2.74)	≤ 3	
143–148	Mouth and pharynx	72	1.76 (1.37–2.18)	≤ 3	
150	Oesophagus	51	1.57 (1.17–2.00)	≤ 3	
161	Larynx	42	2.08 (1.49–2.73)	≤ 3	
162.1	Lung	327	1.57 (1.41–1.75)	76	1.62 (1.26–2.01)
181	Urinary bladder	203	1.19 (1.03–1.35)	12	0.82 (0.41–1.30)

*Obs* observed cases; *CI* confidence interval

**Table 5:** Standardized Incidence Ratio (SIR) for cancer 1985 to 2011 among all Swedish male merchant seafarers and those ever working on tankers across three calendar periods by first sea service (<  1985, 1985–1991, > 1992)

All male seafarers	< 1985		1985–1991		> 1992		Trend *P* value
	Obs	SIR (95% CI)	Obs	SIR (95% CI)	Obs	SIR (95% CI)	
All sites of cancer	2544	1.10 (1.06–1.15)	360	0.86 (0.78–0.96)	317	0.93 (0.83–1.03)	< 0.0001
Lung cancer	300	1.65 (1.47–1.83)	27	1.07 (0.68–1.51)	14	0.76 (0.38–1.19)	0.0014
Urinary bladder cancer	185	1.24 (1.07–1.43)	18	0.84 (0.47–1.26)	15	0.95 (0.51–1.46)	0.1188
Male tanker seafarers							
All sites of cancer	467	1.14 (1.04–1.25)	375	1.06 (0.95–1.17)	99	0.93 (0.75–1.12)	0.07
Leukaemia^a^	19	1.44 (0.84–2.13)	18	1.52 (0.84–2.27)	4	0.99 (0.25–1.99)	< 0.05
Lymphoma^a^	16	1.07 (0.60–1.60)	11	0.80 (0.36–1.31)	4	0.94 (0.23–1.87)	0.50

*Obs* observed cases; *CI* confidence interval

^a^1985–2011 Leukaemia SIR 1.41, 95% CI 1.00–1.86, Lymphoma SIR 0.94, 95% CI 0.64–1.27

There were in total 16 cases of mesothelioma, and all had occurred among seafarers with their first employment before 1985. Eleven of these cases, compared to 4 expected cases, had occurred among ever engine room crewmembers (SIR 2.59; 95% CI 1.18–4.23).

Seafaring men had fewer cases of prostate and thyroid cancer than expected (Table 3).

The analysis on haematologic malignancy amongst men having served on tankers revealed 83 cases in total, which was similar to the expected number (*n* = 78). However, there were more cases of leukaemia than expected (Table 5). The SIR of haematologic malignancy decreased across the three calendar periods, with a significant trend for leukaemia (Table 5).

### Cancer in women seafarers

There were significantly less cases of cancer among women seafarers than expected (938 observed vs. 1127 expected) (Table 2). No position showed an increased risk for total cancer. Lung cancer, however, was significantly more common among women seafarers. Almost every lung cancer case had worked in the catering department (*n* = 81 out of 84 cases) (Table 2) and the majority (*n* = 48) had occurred in the group of seafarers with a first employment before 1985 (data not shown). There were no cases of mesothelioma amongst women (Table 3). Breast cancer, cancer of the uterus, thyroid cancer and leukaemia were all less common than in the general female population (Table 3). The incidence of cancer of the cervix uteri was not increased, which was true also for malignant melanoma or other skin cancer.

Even with a minimum of 5 years of cumulated sea service women seafarers had less cancer cases than expected for all sites together (Table 3). Lung cancer incidence was still increased.

## Discussion

This cohort study with a follow-up for cancer in Swedish seafarers between 1985 and 2011 showed cancer to be more common in male seafarers and less common in women seafarers compared with the general Swedish population. The increased risk of cancer was associated primarily with seafarers with a first sea service before 1985. Higher incidences of lung cancer in both men and women and of urinary bladder cancer in men were noted. Male engine room ratings had an almost doubled risk of lung cancer. Male engine room crewmembers employed before 1985 had an increased risk of mesothelioma. Work on tankers was associated with leukaemia.

The strength of the study is the robustness of data of both independent and dependant variables coming from state-hold registries. One limitation is the fact that the SR lacks information on seafarers that stopped working prior to its digitization in 1985. Another limitation is that we had no knowledge of the type of cargo associated with any sea service, since cargo is not noted in the SR. However, type of ship was noted, which enabled us to analyse associations between blood malignancies and work on tankers. We share the same limitations as other Nordic studies on cancer in seafarers with the exclusion of a foreign workforce, short cumulated sea service time per seafarer and no direct information on lifestyle factors. We also did not have information on other occupations held by the seafarers either before, during and/or after work at sea.

Increased cancer risks were primarily associated with an early start in seafaring (< 1985) and there was a decreasing trend in cancer with later first employments. However, our material was not sufficient to assess cancer risks with later sea starts, since that group of seafarers had a shorter follow-up and the number of cases were few. Increased risks of cancer in seafarers reported in the literature stem primarily from older periods of seafaring up to 1999 at the latest (Pukkala and Saarni 1996; Kaerlev et al. 2005; Ugelvig Petersen et al. 2018; Ugelvig Petersen et al. 2020). Our study pushed this exposure window a bit further, up to 2011. Preferably, other Nordic cohorts can be up-dated with more recent exposure data to corroborate our results of a diminished cancer risk in more recent sea services.

Furthermore, studies on cancer in seafarers generally lack a good measure of exposure to known or suspected carcinogens: there are few on-site measurements, exposures are complex and the length of exposure is seldom known. Therefore, the exposure assessment is usually rather crude and essentially based on ‘department’ or ‘last held position’. The rationale for this method implies that seafarers usually work in one category during his or her whole career at sea (Kaerlev et al. 2005; Oldenburg et al. 2015; Ugelvig Petersen et al. 2018). Except for catering, we have observed that an overlap is common between different job departments or job positions (Eriksson et al. 2020). A risk of misclassification of exposure may thus exist. Using ‘ever’ in our analyses for the independent variables, we sought to reduce this risk of misclassification by not excluding any particular type of sea service.

Lung cancer was the only cancer site where we found an increased risk among women seafarers. We could not corroborate the other findings from the two Nordic studies, which included women seafarers (Pukkala and Saarni 1996; Ugelvig Petersen et al. 2018). Women seafarers in our cohort had worked a substantially less time as seafarers than their male counterparts. However, even when analysing women seafarers with more than 5 years of cumulative sea services, the risk of total cancer was still decreased. The cases were relatively few in number, and we need a longer follow-up for more valid results.

The following argument may indicate an occupational causal factor for cancer in male seafarers: seafarers had more cancer than the general population, the risk increased with a longer cumulated employment (> 5 years) and the risk was either unchanged or rather slightly increased when eliminating cancer diagnosis within 10 years after a first sea service. The last part of this argument stems from the fact that the latency of most cancer types can be considered to be > 10–15 years, leukaemia exempted (Richardson 2008; Manton 2009; Straube et al. 2010; Triebig 2010; CDC 2013; Nadler 2013).

Important confounders when analysing cancer in seafarers may be an elevated use of tobacco and alcohol in seafarers compared to the general population. Although the actual smoking percentage among Swedish seafarers may be as low as 11% and comparable to the Swedish population, it was around 65–70% during the 1960s compared to 50% in the general population at that time (Nilsson 1998; Forsell et al. 2017). For alcohol consumption, we do not have any reliable data for comparison. However, studies on occupational cancer have shown that there often have to be big differences in smoking or other lifestyle factors should they account for a substantially increased risk (Hadkhale et al. 2019). In a sensitivity analysis on occupational risk of larynx cancer, Kriebel et al. found that large differences in smoking and alcohol consumption could only explain 20% or less of the associations found (exposure of metalworking fluids) (Kriebel et al. 2004). Rather, co-exposures to smoking and an occupational carcinogen might add up each separate risk ratio even further (thus not confounding but effect interaction), as is the case with lung cancer, asbestos and smoking (Axelson 1989). The same mechanism has been discussed for cancer of the lungs and the urinary bladder and occupational exposures to PAHs and nitroarenes. In our study, the most elevated SIR of lung cancer was noted in engine ratings, where manual work tasks and hence exposure to occupational carcinogens, would be more common. But we have no indication that ratings would smoke more/have more pack-years than officers—who previously themselves often have been ratings, at least in the group of interest here: seafarers with a start at sea before 1985. In summary, it appears possible that carcinogenic occupational factors for lung cancer and cancer of the urinary bladder exist in seafaring, and that they refer to exposures to PAHs present in oils, soot and exhausts (Hadkhale et al. 2019). The effect size of the risk contribution from occupational exposures rest to be assessed.

Regarding UV radiation, as in other studies, we found an increased risk of lip cancer among male seafarers (Ugelvig Petersen et al. 2018, 2020). But as in the Danish study, we did not find that for malignant melanoma or other skin cancer (Ugelvig Petersen et al. 2018). Asbestos exposure with its long latency time still causes mesothelioma, and as could be expected most of the cases in this study had been working in the engine room. Several other studies have found increased incidence of mesothelioma for male seafarers even if not all of them have been significant (mesothelioma is a rare disease) (Pukkala and Saarni 1996; Ugelvig Petersen et al. 2020).

We found an increased risk of leukaemia for seafaring men that had worked on tankers. Several studies have demonstrated considerate occupational exposures to the leukomogen benzene for the deck crew (Mowe et al. 1977; Moen et al. 1993, 1995; Williams et al. 2005; Kirkeleit et al. 2006; Jacobs et al. 2011). We have shown uptake of benzene and excretion of benzene in alveolar air and urine, together with benzene metabolites, in tanker crewmembers during the mid-1990s (Forsell et al. 2019). Exposure to benzene have probably decreased considerably due to instalment of closed systems for cargo handling and maintenance on board tankers. Decreased occupational exposure appears to have been followed by decreasing incidence of leukaemia, for which we found a significant trend. Smoking is also a risk factor in relation to leukaemia but the seafarers not working on tankers had no increased risk for leukaemia.

The risk of prostate cancer, the most common cancer form in men in the general population, was somewhat decreased in our study. Probably that is a chance finding, since there are conflicting results on the risk of prostate cancer in seafarers (Pukkala and Saarni 1996; Ugelvig Petersen et al. 2020). Regarding thyroid cancer, we have no explanation for the decreased risk in both men and women. One study reported an increased risk of papillary thyroid cancer among male seafarers (Ugelvig Petersen et al. 2020).

## Conclusion

This cohort study of Swedish seafarers active up to 2011 showed an increased risk of cancer in male seafarers with a first sea service before 1985. The risk of total cancer was not increased in women, albeit increased for lung cancer. Several but not all lifestyle-associated cancers were increased in men. The results lend some support to that earlier increased risks of cancer among seafarers are changing to a more normal pattern in modern seafaring.
